# Psychiatric hospitalization rates in Italy before and during COVID-19: did they change? An analysis of register data

**DOI:** 10.1017/ipm.2020.29

**Published:** 2020-05-05

**Authors:** M. Clerici, F. Durbano, F. Spinogatti, A. Vita, G. de Girolamo, R. Micciolo

**Affiliations:** 1 Department of Mental Health and Addiction, ASST of Monza, Monza, Italy; 2 Psychiatric Clinic, University of Milan Bicocca, Milan, Italy; 3 Department of Mental Health and Addiction, ASST of Melegnano e della Martesana, Milan, Italy; 4 Department of Mental Health and Addiction, ASST of Cremona, Cremona CR, Italy; 5 Department of Mental Health and Addiction, ASST of Brescia, Brescia, Italy; 6 Psychiatric Clinic, University of Brescia, Brescia, Italy; 7 UOPEV, IRCCS Istituto Centro San Giovanni di Dio Fatebenefratelli, Brescia, Italy; 8 Department of Psychology and Cognitive Sciences, University of Trento, Trento, Italy

**Keywords:** COVID-19, disaster, hospital admission

## Abstract

**Objectives.:**

To assess admission rates to seven General Hospital Psychiatric Wards (GHPWs) located in the Lombardy Region in the 40 days after the start of Coronavirus disease 2019 (COVID-19) epidemic, compared to similar periods of 2020 and 2019.

**Methods.:**

Anonymized data from the regional psychiatric care register have been obtained and analyzed. The seven GHPWs care for approximately 1.4 million inhabitants and have a total of 119 beds.

**Results.:**

In the 40-day period (February 21–March 31, 2020) after the start of the COVID-19 epidemic in Italy, compared to a similar 40-day period prior to February 21, and compared to two 40-day periods of 2019, there has been a marked reduction in psychiatric admission rates. The reduction was explained by voluntary admissions, while there was not a noticeable reduction for involuntary admissions. The reduction was visible for all diagnostic groups, except for a group of ‘Other’ diagnoses, which includes anxiety disorders, neurocognitive disorders, etc.

**Conclusions.:**

Large-scale pandemics can modify voluntary admission rates to psychiatric facilities in the early phases following pandemic onset. We suggest that the reduction in admission rates may be due to fear of hospitals, seen as possible sites of contagion, as well as to a change in thresholds of behavioral problems acting as a trigger for admission requests from family relatives or referrals from treating clinicians. It is unclear from the study whether the reduction in admissions was contributed to most by the current pandemic or the lockdown imposed due to the pandemic.

## Introduction

It was February 21, 2020, when the first Italian patient, a young male aged 38 years, was found positive for coronavirus disease 2019 (COVID-19) testing. Since then, Italy has seen a dramatic increase in the number of COVID-19+ people and in the number of deaths: according to the Italian National Institute of Health (N.I.H.), which provides a daily update of epidemiological data provided by all 21 Italian Regions, there were 159 107 infected people as of April 16, 2020, and 19 996 deaths caused by the epidemic (Task Force COVID, 2020).

The mental health consequences of the exposure to a natural or man-made disaster can be rather different, as demonstrated by several studies with conflicting results (North & Pfefferbaum, 2013). While it is generally difficult to estimate the psychosocial impact of a disaster on the general population because of sampling problems, difficulties in assessment, type of priorities to be managed, it is easier to assess the effect of a disaster on ‘hard’ indicators of mental health services use: hospital admissions, including both voluntary and involuntary admissions, can be particularly helpful in this regard.

The aim of this contribution is to study the temporal pattern of hospital admissions in four large Departments of Mental Health and Addiction (DMHAs) of the Lombardy Region in Italy: Lombardy was the area most heavily affected by the epidemic. We will compare hospitalization rates in 13 weeks from January 1, 2019, to March 31, 2020: these weeks include 40 days since the diagnosis of the first patient in Italy (February 21, 2020) and the period since the official announcement of the national lockdown, which started on March 11, 2020.

## Italian mental health services: basic figures

According to official figures of the Ministry of Health(Ministero della Salute, 2018), in Italy, there are 134 DMHAs (27 of which are in Lombardy, one for each Health Unit); in 2017, they provided mental health care for 851 189 citizens. Mental health care is available to all, and the costs of care, with few exceptions, are covered by the NHS budget. DMHAs run a full array of community, inpatient, and day-and long-term residential services; in many areas of the country, DMHAs also include addiction services and child/adolescent mental health services. There are 318 General Hospital Psychiatric Wards (GHPWs), with a total of 3,981 beds (average 12.5 beds each), and 22 private facilities with 1,155 beds (even here the cost of hospital stay is covered by the NHS), giving a total of 10.1 acute beds for 100 000 adult population in the whole country. In 2017 (last year of available data), there were 97 276 admissions to the 318 GHPWs, with a total of 1 160 151 hospitalization days and an average length of 12.9 days per admission. The annual rate of admissions per 1,000 population aged 18 and over was 1.9, with some variations across different Regions. In the same year, there were 7,608 involuntary admissions, that is, 7.8% of all admissions to GHPWs.

## Methods

The analysis presented here focuses on admissions to seven GHPWs located in the catchment areas of four DMHAs of the Lombardy Region, notably Brescia, Cremona, Melegnano e della Martesana, and Monza. Table 1 shows the main demographic and service-related characteristics of the 4 DMHAs: they cater for almost 1.4 million inhabitants.

**Table 1.** Catchment areas of the four DMHAs included in the analysis

DMHA	Catchment area POPN ≥ 18 years	Number of municipalities	Number of patients in treatment^a^	Number of GHPWs	GHPW beds
Brescia	484 357	52	8643	2	44
Cremona	189 531	77	3781	2	25
Melegnano Martesana	419 213	42	6500	2	30
Monza	245 145	16	4200	1	20
TOTAL	1 338 246	187	23 124	7	119

GHPWs = General Hospital Psychiatric Wards.

a Overall number of patients with at least one contact with the DMHA in the course of 2019.

The Lombardy Region has been equipped with an automated register since 1997: this register collects a variety of information concerning the regional NHS. Information includes data on patients in contact with public DMHAs, for example, sociodemographic data, ICD-10 diagnoses, treatments received (including prescription of medications), hospital admissions and discharges, treatment settings (e.g. outpatient visits or home visits), use of day-hospital, and stay in residential facilities. For each patient, we linked the above databases via a single identification code, which was automatically converted to an anonymous code to preserve privacy. Through this record linkage process, we were able to identify admission rates to the seven GHPWs included in this analysis.

### Time periods

For the analyses of admission rates over time, we considered admissions between January 1 and March 31, 2019 (period A) and between January 1 and March 31, 2020 (period B); in period A (2019), there were 90 days, while in period B (2020), there were 91 days (leap year). Total and mean daily number of admissions were calculated for each of the 13 weeks of period A and period B (the last ‘week’ of period A consisted of 6 days). We also compared monthly numbers and rates of admissions January–March 2019 and 2020; these rates were calculated as daily rates per 1000 adult residents and then converted into annual rates for the sake of comparison with national data.

### Statistical analyses

Poisson regression analysis was employed to compare admission rates in different periods and/or in different subgroups. Survival analysis techniques were employed to estimate the proportion of subjects discharged over time and to compare the lengths of the hospital stay.

## Results

### Admission rates before and during the COVID-19 epidemic

**Fig. 1.**
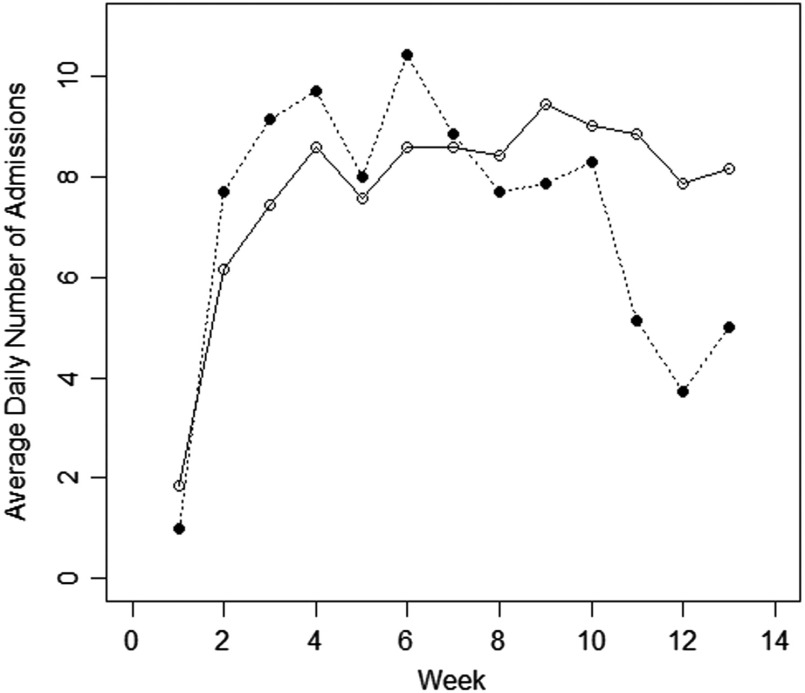
Average daily number of admissions to seven GHPWs in 13 weeks from January 1, 2019, to 31 March, 2019 (solid lines) and in the corresponding weeks from January 1, 2020, to March 31, 2020 (dotted lines).

Figure 1 shows the temporal trends in admissions in all weeks starting on January 1, 2019, and 2020, up to March 31. It can be seen that the fall in admissions started on week 11 of period B (i.e. in March 2020), and in particular, the decline initiated on March 11, when the Italian government declared the general lockdown. Poisson regression shows that admission rates in January 2020 were not significantly different from those found in January 2019 (*p* = 0.18) and that admission rates in February 2020 were not significantly different from those found in February 2019 (*p* = 0.68); on the other hand, admission rates in March 2020 were significantly different from those found in February 2019 (*p* < 0.001).

Analysing the mean daily number of admissions calculated in each of the 13 + 13 weeks considered, it emerges that the largest reduction in percentage (compared to the corresponding week of 2019) was at week 12 (−53%), but was already visible at week 11 (starting on March 11) with −42% and proceeded up to week 13 (last week analyzed) with a −39% reduction (see Table 2); it is worth noting that, even if ‘week’ 13 in 2019 consisted of only 6 days, while week 13 in 2020 consisted of 7 days, the absolute number of admission was greater in 2019 than in 2020 (49 *v.* 35; daily average: 8.17 *v.* 5.0). The average daily number of admissions and the absolute number of admissions per week in 13 weeks January–March 2019 and 2020 are shown in Table 2.

**Table 2.** Average daily number of admissions and total number of weekly admissions in 13 weeks (January 1–March 31) in 2019 and in 2020

	Year	Weeks												
		1	2	3	4	5	6	7	8	9	10	11	12	13
Average daily number of admissions	2019	*1.8*	*6.1*	*7.4*	*8.6*	*7.6*	*8.6*	*8.6*	*8.4*	*9.4*	*9.0*	*8.9*	*7.9*	*8.2*
Total number of weekly admissions		13	43	52	60	53	60	60	59	66	63	62	55	49
Average daily number of admissions	2020	*1.0*	*7.7*	*9.1*	*9.7*	*8.0*	*10.4*	*8.9*	*7.7*	*7.9*	*8.3*	*5.1*	*3.7*	*5.0*
Total number of weekly admissions		7	54	64	68	56	73	62	54	55	58	36	26	35

**Table 3.** Total number of admissions to seven GHPWs in Italy during six index periods and corresponding annual admission rates (per 1000 adult residents) and mean ages at admission. For the sake of comparison with national data, admission rates are referred to a period of 365 days

	Number of admissions						Rates of admissions					
Month	January		February		March		January		February		March	
Year	2019	2020	2019	2020	2019	2020	2019	2020	2019	2020	2019	2020
All admissions	194	221	236	245	265	182	1.70	1.94	2.29	2.30	2.33	1.60
Voluntary admissions	181	212	220	226	245	164	1.59	1.86	2.14	2.12	2.15	1.44
Involuntary admissions	13	9	16	19	20	18	0.11	0.07	0.15	0.17	0.17	0.15
Diagnosis												
*Schizophrenia spectrum disorders*	13	9	15	9	24	15	0.11	0.07	0.14	0.06	0.21	0.13
*Affective disorders*	62	68	69	59	94	42	0.54	0.59	0.67	0.55	0.82	0.37
*Personality disorders*	57	63	67	93	62	48	0.50	0.55	0.65	0.87	0.54	0.42
*Substance use disorders*	42	57	60	58	56	44	0.37	0.50	0.58	0.54	0.49	0.38
*Other diagnoses*	20	24	25	28	29	33	0.17	0.21	0.24	0.26	0.25	0.29
Mean age at admission ( ± SD)	42.8 ± 16.7	42.5 ± 16.5	44.1 ± 15.9	41.0 ± 16.3	44.3 ± 15.5	43.0 ± 16.0	-	-	-	-	-	-

Table 3 shows the absolute number of admissions, patients’ status (voluntary or involuntary admission), diagnoses, and corresponding annual rates in the 3 months of 2019 and 2020. Overall, in March 2020, there was a 31.3% decrease in the number of admissions compared to March 2019 and a 25.7% decrease compared to February 2020. The decrease was significant for voluntary admissions (*p* < 0.001), while the difference was not significant for involuntary admissions (*p* = 0.87). The number of involuntary admissions, evaluated over the all considered period, was fairly constant, with about 16 admissions on average per month. In terms of diagnoses, the reduction was numerically visible for all diagnostic groups, but ‘Other diagnoses’, which includes severe anxiety disorders, neurocognitive disorders, etc. Separate analyses, performed on different diagnostic groups, revealed an important and highly significant reduction (−55%) in admission rates for affective disorders (*p* < 0.001); on the other hand, the decrease in admission rates observed for schizophrenia spectrum disorders (SSD), personality disorders, and substance use disorders did not reach statistical significance (possibly because the sample size for these diagnoses was not large enough). No significant difference in the mean age at hospitalization was found (*p* = 0.242; Table 3).

### Length of stay

The median time of hospitalization was 12 days (half of patients were discharged within 12 days), 1/4 within 7 days, and ¾ within 21 days. There were statistically significant differences (*p* < 0.001) in the length of stay in relation to diagnosis: the lowest median (7 days) was for patients with SSD, while patients with substances use disorders and ‘Other’ diagnoses stayed a median of 10 days; personality disorders and affective disorders had a median of 13 and 14 days, respectively. There was no difference in length of stay between voluntary and involuntary admissions. There was a significant gender difference in median length of stay, with women being in hospital for longer time (13 days) compared to men (11 days) (*p* = 0.023). The median time of hospitalization in March 2019 was 10 days, while in March 2020, it increased to 14 days (*p* = 0.021).

## Discussion

To our knowledge, this is the first report published after the start of the epidemic showing a decrease in psychiatric admission rates during the lockdown period in an area of Lombardy with nearly 1.4 million inhabitants. Two DMHAs involved in this analysis (Brescia and Cremona) cater for two of the most COVID affected areas in the world, with the highest number of deaths.

Our data show that immediately after the lockdown initiated by the Government (active as of March 11), there has been a marked drop in the average daily number of admissions to seven GHPWs included in this study, and this continued up to the end of the index period (March 31, 2020). The reduction was statistically significant for voluntary admissions and was visible for all diagnostic groups, with the only exception for people with anxiety disorders, neurocognitive disorders, etc. The same trend was visible in all sites in the period April 1–27, 2020, when there were 118 voluntary admissions to the seven GHPWs, with a further, statistically significant reduction compared to March 2020 (164 admissions, *p* = 0.007). It is uncertain whether the lockdown imposed by the government caused the main effect as much as the pandemic itself did.

While annual admission rates per 1000 population >18 in January (1.94) and February (2.30) 2020 were numerically higher as compared to the national admission rate recorded in 2017 (1.90, last year available), in March 2020, this rate decreased to a value (1.60) which is 15.8% lower than the national figure. The median length of hospitalization in March 2020 (14 days) was significantly longer than in March 2019 (10 days).

### What are the likely explanations for the reduction in admission rates?’

Which are the factors that can explain this reduction in hospitalization rates in the 40 days after the start of the epidemic in Lombardy? We can suggest some hypotheses:

1. *Fear of contamination and avoidance of hospitals*

Almost all hospital facilities in Lombardy have been overwhelmed by the epidemic, with entire wards diverted to provide care only to COVID-19+ patients, a large proportion of health staff diverted to the care of COVID-19+ patients and many non-emergency visits or surgeries delayed after the end of the epidemic. Mass media have given great emphasis to the high risk of contamination in hospitals, and this fear may have prevented many patients, and their relatives, from asking for hospital admissions, even in clinical situations (such as psychotic disorders) which may usually require inpatient care. Moreover, some people may have been in hospital or at home suffering from COVID-19; others may have been in enforced isolation, and others may have been unable to travel.

2. *Change in thresholds for hospitalization*

Coupled with the fear and avoidance of hospitals, seen as risky places, a change in clinical and behavioral ‘thresholds’ which act as triggers for hospital admission may have occurred. In other words, patients and family members may have become more tolerant during the epidemic, avoiding referrals to hospital facilities for the fear of exposure to the risk of contamination. At the same time, clinicians may have been more cautious in admitting unknown patients or patients at risk for infection or with uncertain recent contacts or movements, in order to preserve the integrity of psychiatric wards, other patients and healthcare professionals from a possible contagion. This may have produced a similar reduction of access to other hospital wards for patients with other medical conditions: interestingly, a report from 15 Italian hospitals located in Northern Italy, comparing the same periods covered in our study, shows a significant decrease in hospitalization rates related to acute coronary syndrome in all sites during the early days of the Covid-19 outbreak (De Filippo *et al.* 2020).

3. *Increase in outpatient activities*

A decrease in admission rates may have been balanced by an increase of outpatient activities. At the moment, we do not have data to confirm or reject such a hypothesis; however, the authors of this contribution have watched a forced reduction in the activities of many community mental health centers in the study catchment areas, partly compensated by an increase in digital communications with patients (telephone calls, videoconferencing, text messaging, etc.). While this increase is an important indication for the future of mental health care in the post-COVID era, it seems unlikely that the marked reduction in admission rates has been accompanied by a parallel increase in outpatient activities.

4. *Decrease in morbidity rates*

A decrease in hospital admission rates, a treatment option generally reserved to most severe clinical conditions, may be due to a true decrease in the incidence of new cases of severe mental disorders, or of relapses for people already in treatment. The short time considered in this analysis makes this hypothesis unlikely. However, reduction or cancellation of all social gathering, where alcohol and drugs are often consumed leading to a variety of emergency presentations, including intoxications, injuries, etc., may also have contributed to a decrease in the incidence of these conditions leading to contact with mental health services.

### Length of stay before and during the epidemic

The median length of hospital stay in March 2020 has increased compared to the previous year. The longer length of stay in March 2020 (COVID-19 period) as compared to the same month in 2019 is probably due to the difficulty in guaranteeing a ‘safe’ return home for inpatients because in March lockdown became universal and stringent. Overall, the parallel reduction of new admissions, with less pressure to discharge inpatients to free beds for new admissions, may have contributed to a change in length of stay.

The shorter length of stay for people with SSD may be explained by the fact that these patients are generally closely monitored in the community and that hospitalization occurs following a decompensation which can often be rapidly resolved leading to discharge. Patients with mood disorders requiring hospitalization may need a longer time to improve, probably also due to the slow onset of response to antidepressant medications. Personality disorders and substance use disorders sometimes pose clinical challenges which can complicate plans for effective community treatment.

### Psychiatric admission rates and large-scale disasters

The study of admission rates to psychiatric facilities has a long history, starting with analyses done in the fifties and in the sixties of admission rates before, during, and after the Second World War, used as an example of large-scale disasters. In Denmark, a study found that in the years 1940–1941, under the German occupation, there was a decrease in psychiatric admission rates, followed by a 50% increase over the 1939 rates in the years 1942–1945 (especially for females), with a return to a flat level in the years 1946–1948 (Svendsen, 1953). The current study reflects the early admission rates following the onset of the COVID-19 pandemic; however, further longitudinal studies are now needed to determine how the admission rates vary as the effects of the pandemic unfold.

Another study analyzed admission rates for schizophrenia during the pre-war period (1936–1939), the war period (1940–1945 inclusive), and the post-war period (after 1945) in several countries (Australia, Canada, Denmark, England and Wales, Finland, Norway, Sweden, Switzerland, and United States) (Dohan, 1966). The author found a marked decrease in admission rates during wartime for schizophrenia, in both genders, in Finland, Norway, and Sweden, and a smaller decrease in Switzerland and Canada. On the contrary, during wartime, there was a marked increase in first admission of males and a slight increase of females with schizophrenia in the USA. While the author examined several hypotheses to explain such changes, his conclusion of a change in admission rates as due to a change in diet and to decreased food supply has not been confirmed by subsequent studies.

Other studies of psychiatric admission rates in the wake of large-scale disasters have been done more recently and show conflicting results. Perhaps the most relevant study in this area was made by Rosenheck and Fontana (2003) who assessed the use of mental health services in New York City and in other metropolitan areas of the United States after the terrorist attacks of September 11, 2001; surprisingly, they did not find any increase in the use of these services, including inpatient admissions. A Swiss study did not find any increase in psychiatric hospitalization rates after an amok attack (with 14 people killed) in a Swiss canton (Hacker *et al.* 2004). Fried *et al.* (2005) assessed use of mental health services in 14 months before Hurricane Floyd in the USA compared to the 12 months after the event: they found a 10% decrease in inpatient admissions for behavioral health reasons. Other authors compared bed occupancy in the month after the Christchurch earthquake (2011) in New Zealand to the pre-earthquake level and also examined bed occupancy for the 18 months following the disaster, and the same was done with admissions (Beaglehole *et al.* 2015). Mean daily admissions fell by 20% in the month after the disaster compared with the mean 30-day rate preceding the earthquake; admission rates remained lower also in the long term. One of the few studies showing an increase in admission rates was conducted in 2011 after the Great East Japan Earthquake; the authors showed an increase in the number of patients admitted to 2 mental hospitals in 4 weeks after the disaster compared to the 4 preceding weeks, but the sample size was very limited (Sakuma *et al.* 2018).

The correlation between use of hospital beds and community services in Australia has been studied over a 7-year period: the authors found a significant reduction in hospital admissions moving from a hospital-centered model to a model of community care (Low & Draper 2005). The work on the local community network and the support for independent life contributed to the significant reduction of acute hospitalizations. Whether this has contributed to a reduction in admission rates in Lombardy, which can benefit from an extensive network of community mental health services, will need to be accurately studied with registry data of outpatient activities. Other studies have investigated the fluctuations in admission rates due to a variety of general and services related factors (Walsh & Daly, 2016).

### Psychiatric admission rates and epidemics

While there have been several studies assessing mental disorders or distress among the general population or health workers during previous epidemics, including SARS and MERS (Lancee *et al.* 2000; Lee *et al.* 2007; Nickell *et al.* 2004; Reynolds *et al.* 2008; Su *et al.* 2007; Wu *et al.* 2009) or among epidemic survivors (Mak *et al.* 2010), we have been unable to trace any studies focusing on changes in psychiatric admission rates before and during past epidemics. The only study focusing on changes in hospital admissions was done in Canada and was aimed at comparing the expected influenza-related hospitalizations in the first 8 weeks of a mild, moderate, or severe pandemic with the actual reduction in the number of hospital admissions in Toronto during the first 8 weeks of the SARS-related restrictions (Schull *et al.* 2006). The authors found that in the first 8 weeks of SARS-related hospital admission restrictions, there was a modest 12% decrease in the overall admission rate, lower than the specific increase due to SARS-related hospitalizations.

### Disasters and psychosocial consequences on the general population

Some ‘instant’ population surveys seem to show major adverse consequences of the COVID-19 pandemic due to the negative effects of social isolation and loneliness because of quarantine and lockdown (Cowan, 2020), these may include an increase of suicide risk, substance abuse, anxiety, and depression and may be related to the need of quarantine and isolation (Brooks *et al.* 2020). At odds with this data, we did find a marked reduction in admissions to psychiatric wards. This may be at least in part related to the model of intensive community care available in Italy, a model which can reduce feelings of social isolation and loneliness of people in treatment at mental health services.

With regard to the psychosocial consequences of the epidemic on general population’s mental health, there is no hard data currently available, but we cannot exclude that it will be available in the future; future studies, conducted with appropriate methodologies, will be necessary to draw definite conclusions.

### Some lessons for the future

DMHAs should prepare plans for a rapid reorganization of mental health services in the case of large-scale emergencies, when patients may particularly suffer due the sudden introduction of restrictive measures. In particular, it seems appropriate to divert more resources to community services, which can provide direct care to most emergencies.

To achieve this, DMHAs need to be better equipped with appropriate e-health technologies and procedures (de Girolamo *et al.* 2020). For instance, DMHAs need to be able to manage online consultations for patients with frequent outpatient visits, which is unfeasible during a crisis; online consultations for patients requiring changes in medications, monitoring, and support for patients living alone at home, suddenly exposed to a marked isolation, and counselling for patients living in families with high expressed emotion should be in place with digital technologies. E-mental health has been rapidly set up in some areas of the country, but this option should be planned and implemented everywhere. The same applies to support families who have children with ADHD or intellectual disabilities.

### Limitations

The limited time available for this contribution made a more comprehensive analysis of registry data impossible; for instance, analysis of outpatient activities may have helped explain the decrease in hospital admissions, which for the time being we could only hypothesize about.

Another limitation is the lack of data on admissions to other medical and surgical hospital wards which may shed light on overall hospital admissions and could suggest that the reduction of psychiatric admission rates should be framed as a more general reduction of hospital activities.
